# The expression of B23 and EGR1 proteins is functionally linked in tumor cells under stress conditions

**DOI:** 10.1186/s12860-015-0073-5

**Published:** 2015-11-17

**Authors:** Donatella Ponti, Daniela Bastianelli, Paolo Rosa, Luca Pacini, Mohsen Ibrahim, Erino Angelo Rendina, Giuseppe Ragona, Antonella Calogero

**Affiliations:** Department of Medico-Surgical Sciences and Biotechnologies, University of Rome Sapienza, Corso della Repubblica 79, 04100 Latina, Italy; Division of Thoracic Surgery, Department of Medical-Surgical Science and Translational Medicine, University Sapienza, S. Andrea Hospital, via di Grottarossa 1035, 00189 Rome, Italy

**Keywords:** EGR1, B23, NPM1, nucleolus, cancer, *Egr-1 −/−* mice

## Abstract

**Background:**

The nucleolus is a multi-domain enriched with proteins involved in ribosome biogenesis, cell cycle and apoptosis control, viral replication and differentiation of stem cells. Several authors have suggested a role for the nucleolus also in malignant transformation. We have recently demonstrated that under specific circumstances the transcriptional factor EGR1 is shuttled to the nucleolus where it functions as a negative regulator of RNA polymerase I. Since this activity is hampered in *ARF −/−* cells, and ARF transcription is regulated by EGR1 while the turnover of ARF protein is under the control of B23, we speculated that some sort of cooperation between EGR1 and B23 might also exist.

**Results:**

In this work we identified a canonical EGR1 binding site on the B23 promoter through experiments of transactivation and in vitro DNA binding assay. We then found that the levels of B23 expression are directly correlated with those of EGR1, and that this correlation applies to several cellular types and to different stress conditions. Furthermore, we showed that EGR1 stability and accumulation within the nucleolus is in turn regulated by B23 through proteasome involvement, similarly to ARF turnover.

**Conclusion:**

Our results highlight EGR1 as a regulator of B23 expression actively playing within the newly discovered nucleolar B23-ARF-EGR1 network.

**Electronic supplementary material:**

The online version of this article (doi:10.1186/s12860-015-0073-5) contains supplementary material, which is available to authorized users.

## Background

During tumorigenesis, cancer cells increase the production of ribosomes to support the higher rate of protein synthesis associated with cancer growth. This is supported by the increase in rRNA synthesis transcribed in the nucleolus by RNA polymerase I, and correlates with adverse prognosis [1–3]. To contrast this mechanism, the products of Rb and p53, two important tumour suppressor genes, negatively interfere with the RNA polymerase I and the assembly of the transcriptional machinery on the rDNA promoter [4, 5]. p53 activation is under direct control of the negative regulator MDM2, which in turn is inactivated by ARF. This explains why ARF is pivotal for triggering the cell-cycle arrest and the apoptotic programme after oncogenic cues [6]. The stability of ARF is significantly increased in cells that overexpress exogenous B23 (known also as nucleophosmin, numatrim, and NOR38). B23 associates with ARF within the nucleolus, delaying its turnover. As a consequence, the inhibition of B23 by shRNA has destabilizing effects over ARF [7]. ARF mutants unable to bind B23 are unstable and functionally impaired [8]. In mouse embryonic fibroblasts (MEFs) lacking both B23 and p53, ARF is mainly found outside the nucleolus and with a shortened half-life, conferring the cells with higher proliferation rates [9]. B23 is an abundant, multifunctional protein present at high amount in the granular region of nucleoli [10]. It is involved in the regulation of ribosome biogenesis, and the control of genome stability and survival in response to a variety of stress stimuli [11–13]. In fact, *B23 −/−* fibroblasts become aneuploid and have increased levels of P53 in a stable form [7]. Conversely, B23 has been shown to induce senescence in normal primary fibroblasts [14] and its overexpression in tumors lacking p53 is known to promote proliferation [15]. The transcription factor EGR1 (early growth response protein 1) is involved in the transcriptional regulation of responses to a wide number of proliferative, differentiation and stress stimuli [16, 17]. In particular, EGR1 regulates the expression of key genes, including p53, TGFB, CDKN1A/p21 and PTEN, involved in the growth and division of cancer cells, [18–20]. *Egr-1 +/−* and *Egr-1 −/−* MEFs bypass senescence and grow as typically immortalized cells. Besides to cover the role as “gatekeeper” of p53-dependent growth regulatory mechanisms [18], EGR1 has been suggested to play a role as tumour suppressor in several tumor types [21–23]. We have recently demonstrated that EGR1 behaves as a negative regulator of RNA polymerase I [24]. EGR1 localizes to the nucleolus and the more it is expressed the less is the 47S pre-rRNA synthesized. ARF is required for the nucleolar localization of EGR1. Indeed, in cells *ARF −/−* such as the NIH-3 T3 Egr1 does not keep the nucleolar localization and the ability to suppress the synthesis of the 47S ribosomal precursor [24]. In view of the functional interaction of ARF with B23, and the fact that EGR1 regulates the transcription of ARF [25] we have hypothesized that EGR1 might extend its control also on B23 expression. Here, we show that EGR1 promotes its stability and the transcription of B23 gene.

## Results

### The expression of nucleolar B23 correlates with the levels of EGR1

**Fig. 1:**
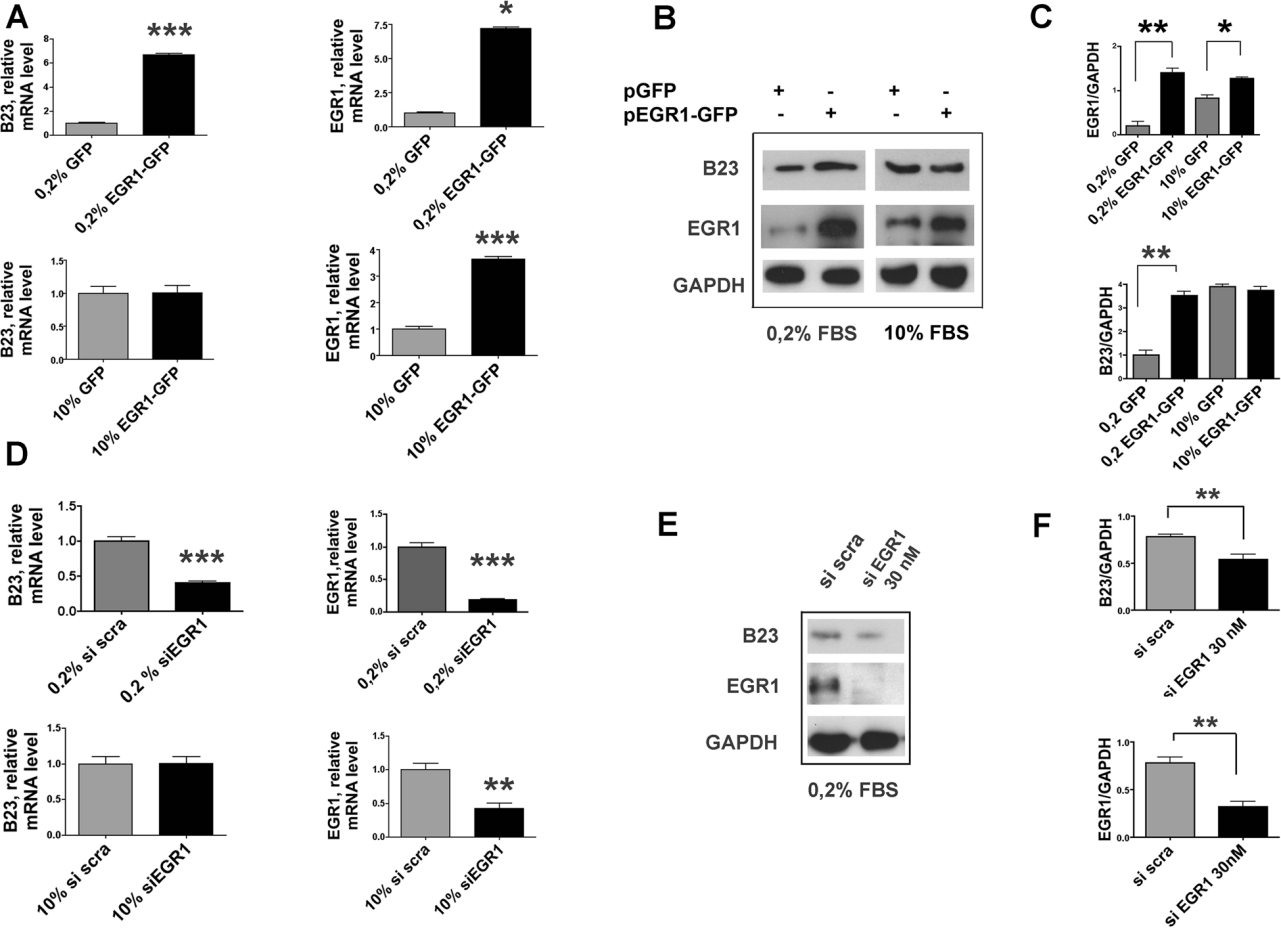
B23 levels are directly correlated to EGR1 under serum deprivation. EGR1 and B23 expression in HeLa cells transfected with a GFP fused full length EGR1 (named EGR1-GFP) or incubated with siRNA specific for EGR1 (named siRNA EGR1). A control empty vector for GFP (named GFP) or a scrambled sequence of the siRNA EGR1 (named siRNA SCRAMBLED) were used for comparison, respectively. Experiments were carried out in medium with either 0.2 % or 10 % FCS. Relative levels of B23 and EGR1 mRNA were quantitated by RT-qPCR in cells transfected with EGR1-GFP or control GFP (Panel **a**), and in cells incubated with siRNA EGR1 or control siRNA scrambled (Panel **d**). Protein signal ratios of EGR1 and B23 to GAPDH were quantitated by densitometry in cells transfected with EGR1-GFP or control GFP (Panel **c**), and in cells incubated with siRNA EGR1 or control siRNA scrambled (Panel **f**), limitedly to serum deprived conditions. Immunoblot of EGR1 and B23 proteins from a representative experiment (Panel **b** and **e**). Data are the mean +/− s.e. of three independent experiments. Comparisons were performed by *t*-test. Significant results are highlighted with asterisks (*p < 0.05; **p < 0.01; ***p < 0.001)

To verify our hypothesis we first monitored the effects on the expression of B23 in HeLa cells by either increasing or reducing the levels of EGR1. After transient transfection of the *pEGFP-EGR1* expression vector, showed a six-fold increase of B23 mRNA in low serum conditions (Fig. 1a). No changes were observed under culture conditions with 10 % FBS. Accordingly, lowering the EGR1 mRNA levels with siRNA specific to EGR1 sequence, we observed a significant reduction of B23 mRNA levels compared to cells treated with non-specific (scrambled) siRNA (Fig. 1d upper panel). Here again, the inhibitory effect on B23 mRNA synthesis was observed only in cells cultured in low serum conditions, and not in presence of 10 % FBS. Similar changes were observed with the B23 protein synthesis under the same experimental conditions. In summary, in condition of serum deprivation the B23 mRNA and protein levels increased after EGR1 overexpression and decreased after EGR1 silencing.

To verify that the above results in HeLa cells are not dependent upon the specific cellular context, we replicated the experiments with a primary culture of human lung tumor and a glioblastoma derived established cell line, U87MG and 293 T (Additional file 1: Figure S1). In all these cultures the B23 mRNA and protein underwent variations in their levels similar to those observed in HeLa under the same experimental conditions. We conclude that the levels of B23 expression are strongly influenced by EGR1 under lower FBS concentration, a known stress inducing condition.

**Fig. 2:**
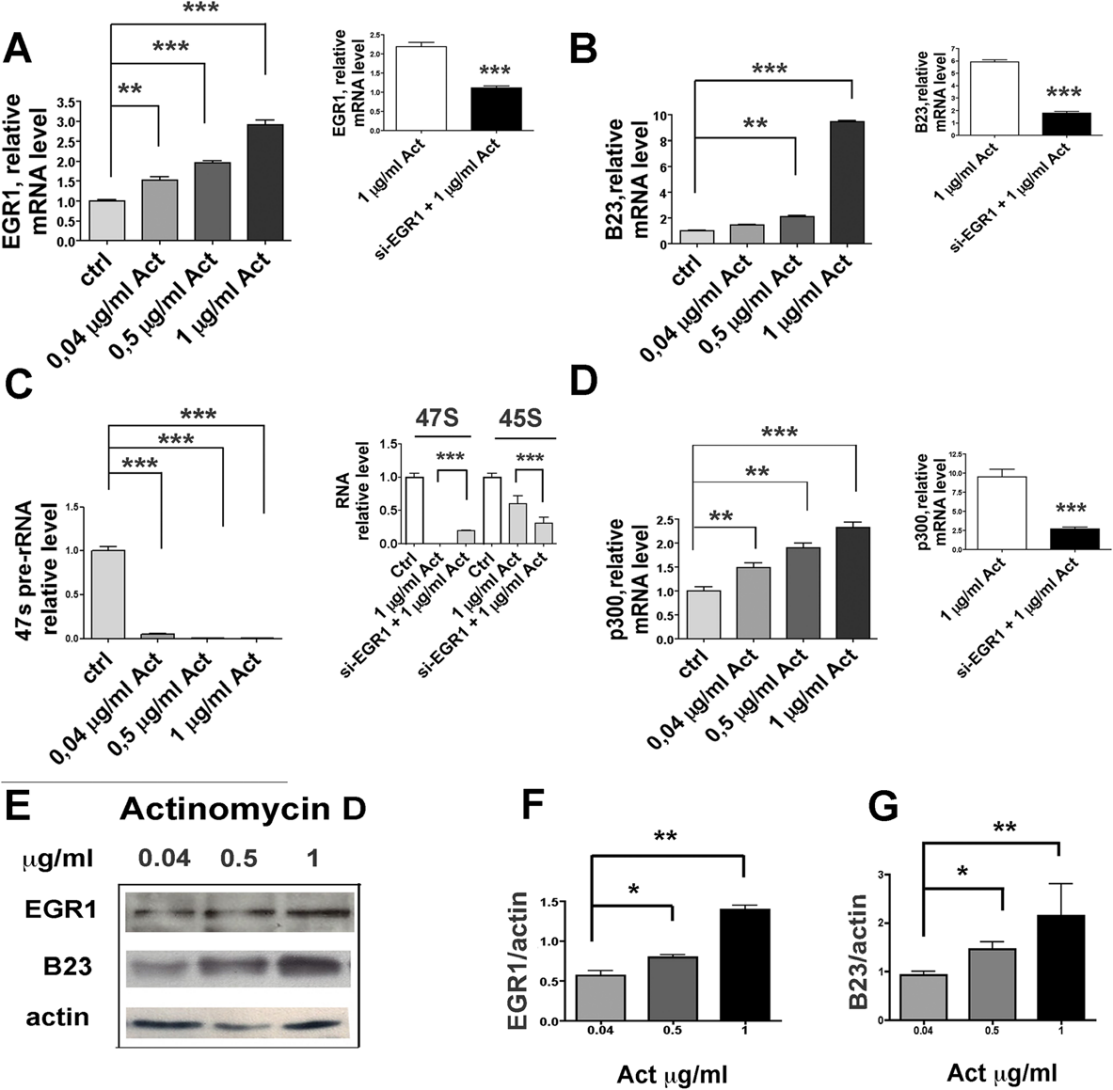
Actinomycin D induces EGR1 and B23. Relative mRNA levels of EGR1, B23, 47S,45S pre-rRNA and p300 (the last two are known gene targets of EGR1) expressed in HeLa cells treated with Actinomycin D for 1 h at 37 ° C (Panel **a** to **d**). The smaller inserts in the panels compare the effect of siRNA EGR1 plus Actinomycin D to that of Actinomycin D alone. Quantitative evaluation of EGR1 and B23 proteins under the effect of Actinomycin D, together with a representative immunoblot, are shown in Panel **e** to **g**. Data are the mean +/− s.e. of three independent experiments. Comparisons were performed by one way ANOVA. Significant results are highlighted with asterisks (*p < 0.05; **p < 0.01; ***p < 0.001)

To investigate whether EGR1 can influence the expression of B23 under different stress conditions, we sought to examine their response to actinomycin D, a genotoxic agent known to inhibit the RNA Polymerase I and II activity and to induce DNA damage [26]. The levels of EGR1 and B23 mRNA and protein showed a step-wise increase when cells were treated with increasing concentrations (0.04, 0.5, and 1 μg/ml) of actinomycin D (Fig. 2a-b-e-f-g). Also the expression of p300, a specific EGR1 target gene, increases, whereas the synthesis of 47S and 45S is strongly inhibited, as shown in Fig. 2c-d. Pre-treatment with EGR1 silencing siRNA abolished the response of B23 and p300 genes and restored the production of 47S rRNA (Fig. 2c-d). Finally, we asked the question of whether the B23 protein would translocate to the nucleoplasm in response to Pol I-induced transcription repression, as suggested by Yao Z. et al. [27]. We confirm that in our experimental conditions actinomycin D causes the translocation of B23 to the nucleoplasm (Additional file 2: Figure S2).

### EGR1 binds to B23 promoter

Since the levels of B23 expression vary in response to EGR1, we hypothesized that as a transcriptional regulator it might control the B23 expression by interacting with the promoter. To verify this hypothesis, we first searched the *B23* promoter (NCBI, Accession: NG_016018) for the presence of canonical sequences that were recognized and bound by-EGR1.

**Fig. 3:**
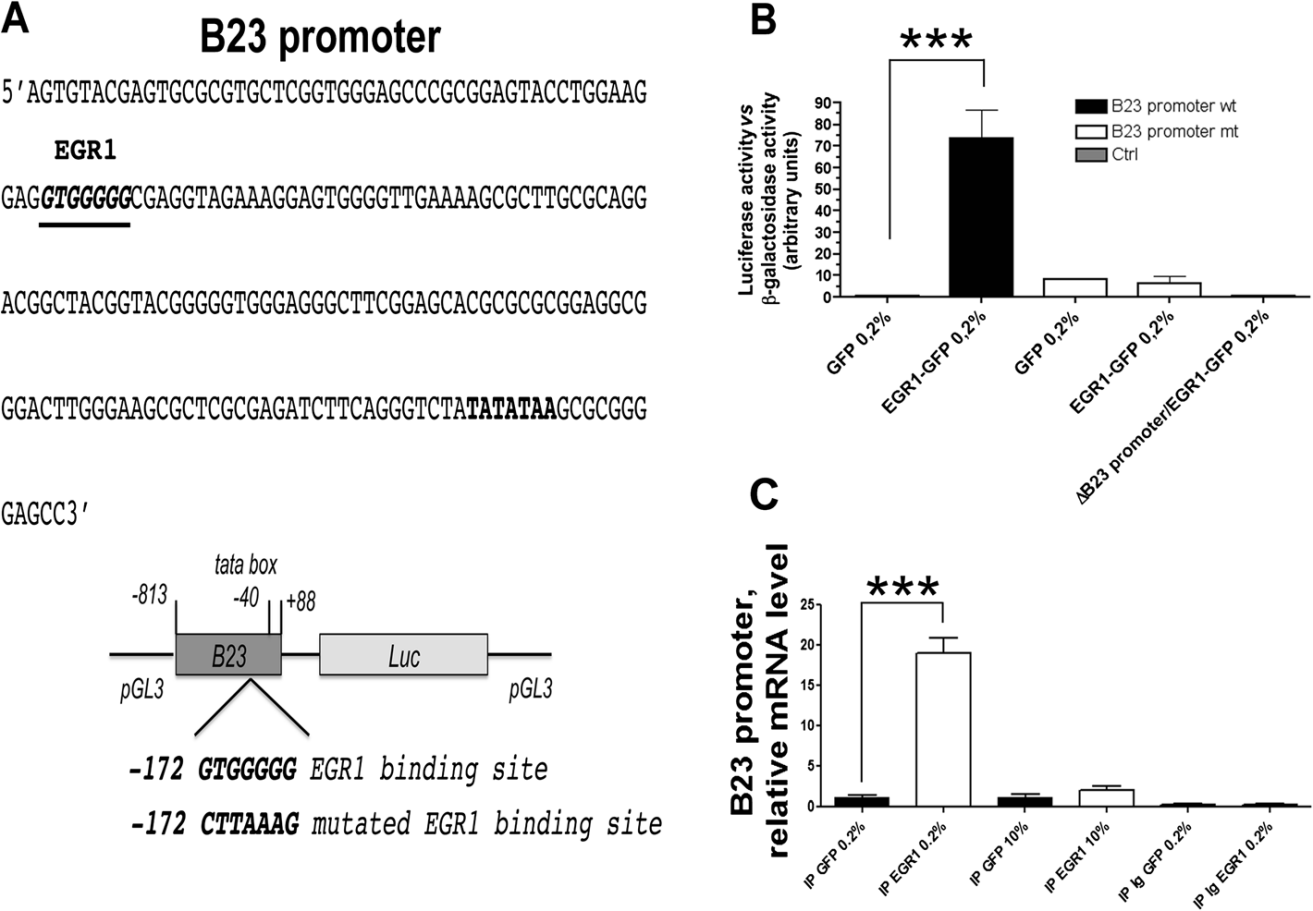
Identification of the EGR1-binding sequence required for B23 promoter induction. **a** The human B23 promoter sequence, partial. An EGR1 binding motif is underlined at position −172 from the TATA box. A schematic representation of the *WT* sequence of *B23 promoter* and a copy with a mutated EGR1 binding site cloned upstream of *Renilla luciferase* gene (*pB23-Luc*, *pB23-mut-Luc*). **b** The luciferase assay in HeLa cells transfected with EGR1-GFP or the control GFP in combination with either of the two luciferase vectors (*pB23-Luc* or *pB23-mut-Luc*) plus a beta-galactosidase vector for normalization of transfection efficiency. All experiments were done under serum deprivation conditions. **c** Real time PCR of HeLa cells extracts either transfected with full length EGR1 or control GFP were incubated with DNA fragments amplified from the promoter sequence of B23 gene, in presence or absence of anti-EGR1 antibody

By-bioinformatics-approach (http://bip.weizmann.ac.il/index.html) one of such elements was found at −172 bp to the TATA box (Fig. 3a). To establish the function of the putative EGR1 binding site, we cloned a partial sequence of the *B23* promoter (4328 to 5240 bp) from HeLa DNA and inserted into a luciferase reporter gene to assay the responsiveness to the activity of EGR1. As above, EGR1 was exogenously expressed following transient transfection of the *pEGFP-EGR1* expression vector. All experiments were carried out in HeLa cells cultured in low serum conditions (0.2 % FBS). The *B23* minimal promoter carrying the sequence of the EGR1 binding site found in HeLa was successfully transactivated by the exogenously expressed EGR1, providing high levels of luciferase activity (Fig. 3b). No luciferase activity was detected when the B23 minimal promoter was deleted of the sequence or carried a mutated version of the EGR1 binding site and tested in the same conditions as above (Fig. 3a-b).

In vitro DNA binding assays were performed to establish whether EGR1 specifically associated with the minimal B23 promoter. We incubated DNA fragments of the promoter sequence with anti-EGR1 antibody in presence of extracts of HeLa cells either transfected with full length EGR1 or EGFP [24]. Cells were cultured either in low serum conditions or at 10 % FBS. An abundant (about twenty-fold) enrichment of promoter sequences was obtained by RT-qPCR (Fig. 3c-d) and PCR (Additional file 3: Figure S3) only with extracts of cells transfected with EGR1 and cultured in low serum. No statistically significant differences were detected in 10 % FBS. Furthermore, when the PCR was performed with oligonucleotides specific for a region located upstream the minimal *B23* promoter no amplification products were obtained (data not shown).

### B23 expression is downregulated in the brain of *Egr1* −/− mice

**Fig. 4:**
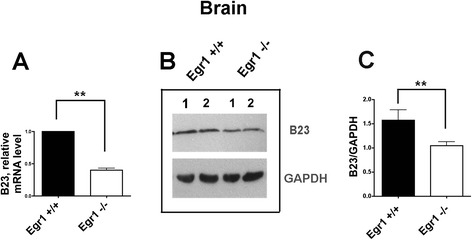
B23 is downregulated in *EGR1 −/−* mouse brain. **a** B23 mRNA and **b**-**c** protein levels in *EGR1 +/+* and *EGR1 −/−* mice. Whole brain extracts (80 μg) were obtained from nine wt and EGR1 −/− female three month old mice. The EGR1 protein was quantified as relative to that of GAPDH taken as control. Comparisons were performed by *t*-test. Significant results are highlighted with asterisks (**p < 0.001)

In order to gain further evidence about the role of EGR1 as a regulator of *B23* expression, we compared the levels of B23 mRNA and protein from brain of *Egr1* −/− with *Egr1 +/+* mice. Since EGR1 is present at high levels in mouse and human brain [28] but not, the levels of B23 expression should likely be different in the brain of wild-type compared to *Egr1 −/−* mice, in case B23 were regulated by EGR1. A significant reduction of B23 mRNA and protein was detected in the brain of *Egr1* −/− mice (Fig. 4).

### B23 increases the EGR1 steady-state level of expression

**Fig. 5:**
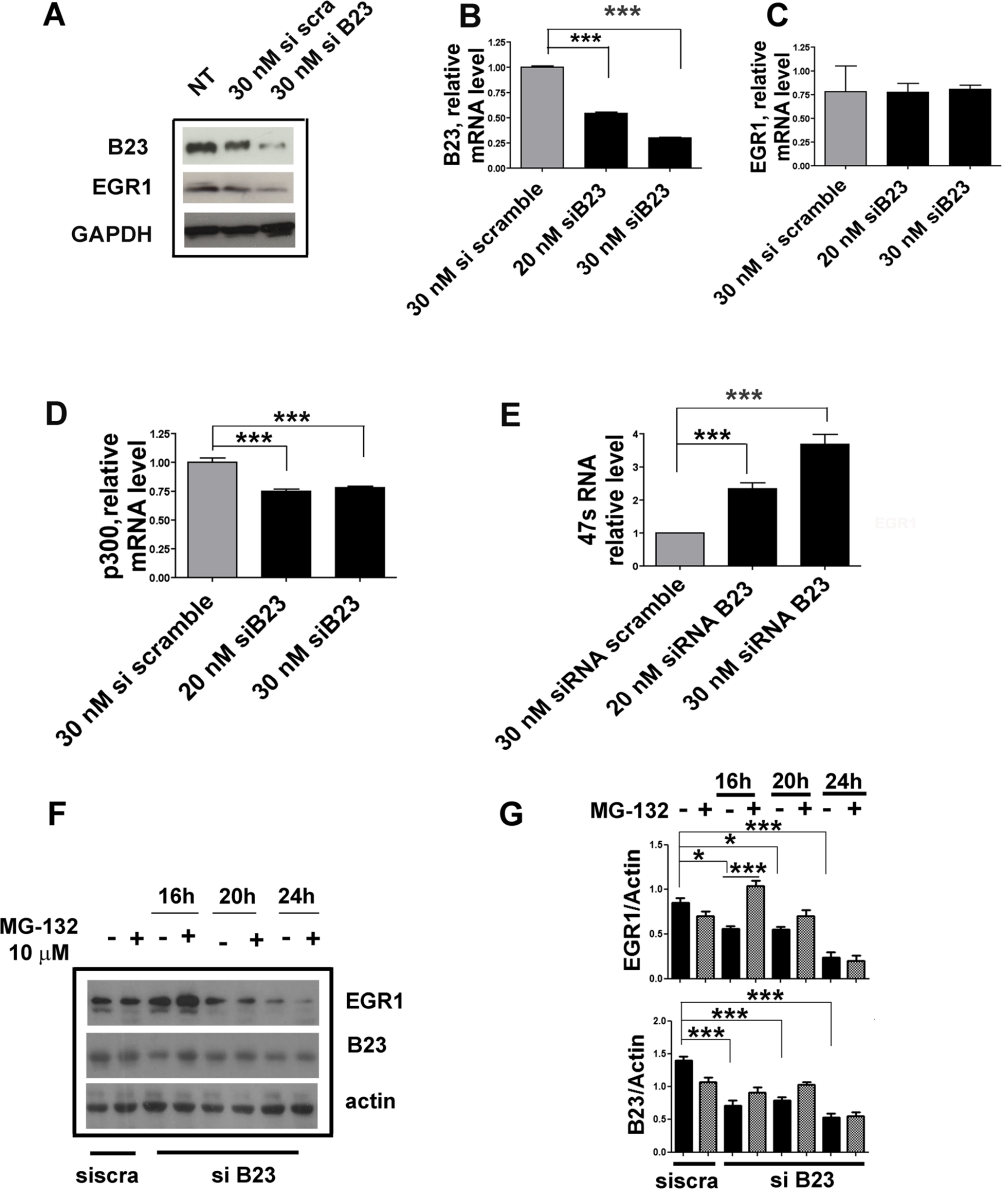
EGR1 protein level is linked to B23. Analysis of EGR1 after B23 siRNA in HeLa cells grown at 0.2 % FBS. **a** Immunoblotting of EGR1 and B23 in whole extracts of HeLa cells after B23 siRNA. EGR1 protein significantly decreases following endogenous B23 silencing at 30 nM. **b**-**c**-**d**-**e** Real time PCR analysis of B23, EGR1, p300 mRNA and 47S pre-rRNA under B23 silencing. The level of expression of EGR1 remained unchanged (**c**). As expected p300 mRNA decrease (**d**) while the 47S rRNA levels increase levels (**e**). **f**-**g** Immunoblotting of EGR1 under MG-132 treatment after B23 siRNA for 16, 20 and 24 h. The level of EGR1 protein is restored during B23 silencing after treatment with proteosome inhibitor at 10 μM for 16 h at 37 ° C. Comparison tests were performed by one way ANOVA and significant results are highlighted with asterisks (*p < 0.05; **p < 0.01; ***p < 0.001)

B23 plays an important role in cell growth by regulating the function of proteins such as ARF and p53 [29]. This is accomplished by delaying the turnover of these proteins, thus contributing to the activation of suppressor activities aimed to the control of cell cycle. In the case of ARF, a physical interaction with B23 has been demonstrated which protected ARF from proteasomal degradation [29]. We asked the question of whether a similar interaction might exist also between EGR1 and B23. If B23 stabilizes EGR1, then a fall in the B23 synthesis would likely translate into a change in the detectable levels of EGR1, due to a loss of stability. To test this hypothesis, we treated the HeLa cells with 30nM B23 specific siRNA, and monitored the expression of both genes EGR1 and B23. We show that following a decrease of B23 mRNA and protein, the levels of EGR1 mRNA remained unchanged (Fig. 5c). However, the levels of EGR1 protein detected by immunoblotting were significantly lower (Fig. 5a-b). We also confirm that under the same experimental conditions the level of 47S pre-rRNA increased whereas the level of p300 diminished, both significantly (Fig. 5d-e). To investigate whether the lower levels of EGR1 protein following B23 silencing were the result of a proteasome-dependent degradation, we tested in HeLa cells the effect of the proteasome inhibitor MG-132 (at the concentration of 10 μM) on the stability of EGR1 during B23 silencing after 16, 20, and 24 h. Only after 16 h from the treatment the levels of EGR1 did actually increase (Fig. 5f-g). These experiments clearly suggest the partial involvement of proteasome machinery on EGR1 turnover, and that, EGR1 stability and accumulation within the nucleolus is likely regulated by B23.

## Discussion

B23 (also known as NPM1, nucleophosmin, nucleoplasmin, NO38, and numatrin) is the best studied member of the NPM family of chaperones, a highly conserved protein found in humans, rodents, chicken and fish. Human *B23* is mapped to chromosome 5q35 and is made up of 12 exons. B23, first isolated from *Xenopus laevis* oocyte extracts [30] is a histone storage protein affecting many intracellular processes such as: i) the rate of ribosome synthesis by binding to pre-ribosomal complexes; ii) DNA duplication and transcriptional regulation; iii) nucleo-cytoplasmic shuttling and assistance to small basic proteins such as REV, REX, TAT and p120 for transport to the nucleolus [31–34]. Within the nucleolus, B23 binds to ARF. The interaction with B23 increases ARF stability by interfering with the action of the ubiquitin-proteasome system.

In a recent publication, we have shown that the transcriptional factor EGR1 also localizes to the nucleolus and acts as negative regulator of RNA polymerase I activity, similarly to p53 [24]. Both B23 and EGR1 are activated and expressed under at least two different stress stimuli, i.e., after UV exposure and H_2_O_2_ treatment [35, 36]. We do not know whether the two genes are independently. To investigate this hypothesis, we conducted a series of experiments where HeLa cells grown in serum deprived conditions and treated either to express high levels of exogenous EGR1 or to silence the expression of the endogenous EGR1 gene, were examined for changes in the localization and the expression of B23 mRNA and protein, and in the regulation of 47S and 45S pre-rRNA synthesis. The above results show that both pre-rRNAs (47S and 45S) have a rise in their levels when EGR1 is silenced. Though very preliminar this observation leading us to hypothesize that the regulation of 47S could be not an abortive transcription. However, further investigations will help us to clarify this point. Here we show that in HeLa cells the levels of B23 mRNA and protein increase after EGR1 overexpression, and decrease after EGR1 silencing. We know that this correlation is not cell specific. In fact, very similar results were obtained also with established glioma cell line and a primary culture of lung adenocarcinoma. In addition, the same effect can be assumed *in vivo* for the brain of B23 by comparing the expression levels of B23 in the brain of EGR1 −/− with that from *EGR1 +/+* mice. An investigation of the human and mouse *B23* promoter nucleotide sequence allowed the recognition of a potential EGR1 binding site, which proved to be effective in a reporter gene transactivation assay. The association of EGR1 to the *B23* promoter was further demonstrated by chromatin immunoprecipitation (ChIP). It has been shown that following DNA damage B23 binds to chromatin [37] and to other proteins as well, such as GADD45 [38], the retinoblastoma protein pRB [39], PARP1 and PARP2 [40]. They are all intimately linked to the maintenance of DNA structure, replication or repair. Following treatment with actinomycin D, nucleolar B23 protein undergoes a rapid translocation to the nucleoplasm [41]. Here we confirm these data and find that the levels of both EGR1 and B23 increase with different concentrations of the drug. However, if cells are treated under conditions inhibiting the expression of EGR1, then the levels of B23 remain constant. One of the mechanism of actinomycin D is inhibit the transcription by intercalating with DNA, in particular at the GC-rich RNA Polymerase I genes. In our experiments EGR1 seem to facilitate the action of genotoxic drug. We have already demonstrate in a previous paper that EGR1 bind UBF protein and the formation of this proteins complex could help the action of Actinomycin D on the inhibition of RNA Polymerase I gene. This is only one of the possible scenario further experiments will help to clarify the role of EGR1 under actinomycin action. These data further suggest a direct role for EGR1 in regulating the expression of B23, and that the correlation between EGR1 and B23 is maintained under several conditions of cellular stress. Several studies have demonstrated the involvement of B23 in the p53 tumor suppressor pathway. Colombo et al. found that B23 when overexpressed promotes cellular senescence in fibroblasts and regulates the stability of p53 through direct interaction [14]. Further, in response to nucleolar stress, B23 does promote the stabilization of ARF protein [25]. In view of the multiple interactions that link B23 to the above molecules and likely to EGR1, we finally studied the effects of B23 over EGR1. Indeed, by inhibiting the expression of B23 we observed a reduction of EGR1 protein levels but not of EGR1 mRNA. Since MG-132, a proteosomal inhibitor, can reduce this effect we speculate that B23 might have a role for keeping adequate levels of the intracellular concentration of EGR1 following stress conditions.

## Conclusion

In summary, these findings provide the evidence that B23 is regulated by EGR1 under stress conditions, and highlights EGR1 as an important link of the ARF-B23-rRNA nucleolar network [42], regulating ribosome biogenesis and promoting growth arrest.

## Methods

### Cell lines, primary tumour cell and mice

The cell lines, uterine cervix cancer HeLa (ATCC CCL2), human embryonic kidney 293 T (ATCC CRL-1573) and glioma U87MG (ATCC HTB-14) were grown in DMEM supplemented with 1 % nonessential amino acids, 1 % L-glutamine, 100 IU/ml penicillin, 100 IU/ml streptomycin and FBS (Sigma-Aldrich St. Louis, Mo, USA) 10 % or 0.2 % (serum starvation) at 37 °C in 5 % CO_2_ humidified atmosphere in air. The cell lines used were obtained from the American Type Culture Collection, Rockville MD, USA. The primary lung tumour cells were grown in DMEM-F12 [43]. The research protocol was approved by ethic committee of S. Andrea Hospital, University of Roma Sapienza. The study was conducted using C57BL/6 wild-type and Egr1 deficient mice (*Egr-1−/−*) [44]. Animals were housed four per cage. All the experiments were performed in accordance with the EU Directive 2010/63/EU for animal experiments and approved by ethic committee of Department of Medico-Surgical Sciences and Biotechnologies, University of Roma Sapienza.

### RT-qPCR

RNA extraction was performed after EGR1 silencing, B23 silencing and EGR1 overexpression into HeLa cells. For silencing, EGR1 and B23 pre-designed siRNA (Invitrogen, Grand Island, NY USA, 4390822) or scrambled sequence RNA oligonucleotide (Negative Control siRNA 4390846) were transiently transfected at 20, 30 nM or 60 nM using High-Perfect Transfecting Agent (as indicated in the figures) (Qiagen, Valencia, CA) following the Qiagen protocol. The treatment with actinomycin D (Sigma) was performed for 1 h at 37 ° C at the three concentrations indicated in Fig. 2. The treatment with the proteasome inhibitor MG-132 (Sigma) was performed at 37 °C for 16, 20 and 24 h during B23 silencing. For EGR1 overexpression *pGFP-EGR1* expression vector [24] was transfected using lipofectamin (Invitrogen, Grand Island, NY USA). To analyse the levels of B23 *in vivo*, total RNA was extracted from female mice of wild type and *Egr1* −/− mice. Total RNA extraction and cDNA preparation were accomplished using RNeasy (Qiagen, Valencia, CA) and Superscript III RT (Invitrogen, Grand Island, NY USA) according to the manufacturer’s recommendations. For the silencing and overexpression experiments we used the protocols described previously [24]. Quantitative RT-PCR was performed using Fast SYBR Green Master mix and the StepOnePlus real-time PCR system (both from Applied Biosystems). Each experiment was performed in triplicate. Experiments were independently repeated three times. Gene expression levels were quantified from real-time PCR data by the comparative threshold cycle (CT) method using 18S as an internal control gene. The following gene-specific primers were used: B23: FW 5'-AGAAAAAGCGCCAGTGAAGA-3’, REV 5’-TGGTGTTGATGATTGGTTTTGA-3’ (for human gene), FW 5’-GGGGGTACCGCTTTCTTTCAGGAGG-3’, REV 5’-CCGCTCGAGGGCACGCACTTAGGTA-3’ (form mouse gene); EGR1: FW 5’- AGCCCTACGAGCACCTGAC-3’ and REV 5’-GGTTTGGCTGGGGTAACTG-3’; 47S: FW 5’-TGTCAGGCGTTCTCGTCTC-3’ and REV 5’-GAGAGCACGACGTCACCAC-3’. 18S: FW 5’-GCAATTATTCCCCATGAACG-3’ and REV 5’-GGGACTTAATCAACGCAAGC-3’; GAPDH: FW 5’-AGCCACATCGCTCAGACAC-3’ REV 5’-GCCCAATACGACCAAATCC-3’; p300: FW 5’-GGTCAAGCTCCAGTGTCTCAA-3’ REV: 5’-GGGACTTAATCAACGCAAGC-3’. Each experiment of transfection, immunoblotting and qPCR were repeated three times in triplicate.

### Immunoblotting

Immunoblotting analysis was performed using whole extracts (40 μg) from HeLa cells after silencing and overexpression of EGR1 [23], or B23 silencing. Total extract (40 μg) were prepared from subconfluent cultures by resuspending cells in RIPA-Buffer (20 mM Hepes, pH 6.8, 5 mM KCl, 5 mM MgCl_2_, 0.5 % NP-40, 0.1 % sodium. Furthermore whole extracts (80 μg) were obtained from brain of nine of wild type and *Egr1 −/−* female mice of three months using RIPA buffer (20 mM Hepes pH 6.8, 5 mM KCl, 5 mM MgCl_2_, 0,5 % NP-40, 0,1 % sodium deoxycholate, protease inhibitor (Sigma), 0.1 mM phenylmethylsulfonyl fluoride) using a Dounce homogenizer. After incubation for 30 min at 0 ° C the lysate was centrifuged at 10000 rpm × 15 min 4 ° C. The samples were loaded on SDS-PAGE gel at 8 % or 10 % of acrylamide (Sigma 29:1) and blotted on PVDF (Biorad) using a semidry apparatus (Biorad). After blocking in 5 % dry milk the membrane was incubated with the appropriate antibody. The primary antibodies used are: rabbit polyclonal anti-EGR1 (sc-101, Santa Cruz Biotechnology), mouse monoclonal antibody anti-B23 (ab10530, Abcam), rabbit polyclonal antibody anti-GAPDH (2118, Cell Signaling Technology Biotechnology) and monoclonal antibody anti-actin (sc-47778, Santa Cruz). The secondary antibodies used for western blot are anti-mouse and anti-rabbit (GE Healthcare Bio-Sciences, Piscataway, NJ, USA). After ECL assay (GE Healthcare) the membrane was incubated with film specific for protein detection (Kodak).

### Luciferase and β–galactosidase activity assay

#### Cloning of the B23 minimal promoter

To amplify the proximal B23 promoter region, genomic DNA from HeLa cells was used as template. The primers used are: FW 5’-GGGGGTACCGCTTTCTTTCAGGAGGAAT-3’ REV 5’-CCGCTCGAGGGCACGCACTTAGGTAG-3’. To verify the specificity of the PCR amplification, the fragment obtained was excised from agarose gel and isolated using DNA gel extraction kit (Millipore). The PCR product (region 4328 bp to 5240 bp of B23, NPM1, numatrin sequence; Accession: NG_016018) was *KpnI/XhoI* digested and cloned into the *pGL3* basic luciferase vector (Promega). The correct sequence was checked by direct sequence (Applied Biosystem). Site-specific mutations in the EGR1-binding sites within the *B23* promoter were made with the QuickChangeII Site-Directed Mutagenesis Kit (Stratagene, La Jolla, CA). Briefly, primer sequences used to generate point mutations are: FW 5’- GGAAGGAGGCTTAAAGGAGGTAGAAAGGAGTG-3 REV 5’-CACTCCTTTCTACCTCCTTTAAGCCTCCTTCC-3’. PCR reaction conditions were: 95 °C for 1 min followed by 18 cycles at 95 °C for 50 s, 60 °C for 50 s, 68 °C for 5 min, then 68 °C for 7 min. Following the PCR reaction, the mix was digested by incubating with D*pn I* for 1 h at 37 °C. Clones of mutated plasmids were replicated in bacteria and screened for the correct mutation by DNA sequencing (Applied Biosystem). *Luciferase assay*. HeLa cells were seeded into 6-well plates and transfected with 0.3 μg trans-activator plasmid *pEgr1-GFP*, 0.3 μg *pB23-Luc* or 0.3 μg *pB23-mut-Luc*, plus 0.5 μg of β-galactosidase vector. At 24 h post-transfection, the levels of luciferase activity were measured sequentially from a single sample using the Luciferase reporter assay system (Promega) with a luminometer (TD-20/20 Turner Design). Measurement of luciferase enzyme activity was assayed on whole-cell extract performed using 20 μl of lysate cleared and 100 μl of Luciferase assay Reagent. The luciferase values were normalized to β-galactosidase activity and protein content. The experiments of luciferase assay were repeated three times in triplicate.

### Chromatin immunoprecipitation (ChIP)

HeLa cells were transfected with full length EGR1 expression vector *pEGFEGR1* or *pEGFP* empty vector using lipofectamine 2000, fixed 48 h after transfection with 1 % formaldehyde for 15 min at room temperature and the reaction stopped by addition of 125 mM glycine for 5 min. The other steps of the ChIP experiments were performed according to the manufacturer’s instructions (Magna ChIP, Millipore). The extracts were immunoprecipitated with anti-EGR1 (4153, Cell Signaling Technology) (24). The samples obtained were analysed by RT-qPCR (Applied Biosystem) and standard PCR (Additional file 4: Material and Methods) using specific couples of oligonucleotides for B23 promoter. Primers used for PCR and real time analysis are: FW 5’-TCGAGGTGCTCTCTGGCTCAT-3’, REV 5’-TGCATAATGGCGTCGGCAG-3’ REV 5’-TGCATAATGGCGTCGGCAG-3’.

### Statistical analysis

Each experiment was repeated at least three times. Statistical comparisons were performed using Student’s *t*-test and a one-way analysis of variance (ANOVA).

This research was supported by MIUR (PRIN 2011 Prot. 2010 ZEJWN_007) and University of Rome Sapienza Ricerche Ateneo 2011–2014 to AC; Ateneo 2014 Prot. N. 0067282 to DP.

## Additional files

Additional file 1: Figure S1. EGR1 levels are directly correlated to B23 expression in primary culture of lung adenocarcinoma tumor, kidney and glioma cell lines under serum deprivation. In all these cultures the B23 mRNA underwent variations in their levels similar to those observed in HeLa under the same experimental conditions. B23 synthesis at 0.2 % FBS is significantly depressed following endogenous EGR1 silencing with 60 mM specific siRNA for lung adenocarcinoma (panel A), 30 nM specific siRNA for U87MG (panel B) and 20 nM specific siRNA for 293 T (panel C). The B23 expression does not change in scramble siRNA control. The data shown are the mean of three independent experiments. Comparison tests were performed by one way ANOVA. (** < p 0.01 *** < p 0.001). (PSD 3175 kb) Additional file 2: Figure S2. B23 nucleoplasm translocation in HeLa cells after Actinomycin D treatment. Immunofluorescence analysis of B23 expression after actinomycin D treatment. (A) Control and (B) HeLa cells treated (0,4 μg/ml) of HeLa cells for 1 h at 37 ° C. The images were taken by under a 40X objective with immunofluorescence microscropy (LEICADM4000B). (PSD 5029 kb) Additional file 3: Figure S3. PCR analysis of ChIP samples. Analysis on agarose gel of PCR amplification products of B23 promoter immunoprecipitated with antibody to EGR1 from extracts of transfected HeLa cells at 0.2 % FBS transfected with full length EGR1. (PSD 10140 kb) Additional file 4:
**Materials and Methods: Immunofluorescence and Standard PCR.** (DOCX 16 kb)

## Abbreviations

EGR1: early growth response protein 1
ARF: alternative ready frame
ChIP: chromatin immunoprecipitation
