# Supplementary material for: The expression of B23 and EGR1 proteins is functionally linked in tumor cells under stress conditions
Source: BMC Cell Biol. 2015 Nov 17;16:27. doi: 10.1186/s12860-015-0073-5 (PMC4650859; doi:10.1186/s12860-015-0073-5)
Supplement: Additional file 4: — Materials and Methods: Immunofluorescence and Standard PCR. (DOCX 16 kb) [file 12860_2015_73_MOESM4_ESM.docx]

**Supplementary Material and Methods**

**Immunofluorescence**

The cells were cultured in DMEM in 10% FBS for 18h on Nuc Lab-Tek. Transcription of rDNA genes was inhibited by supplementing the medium with 0.04 μg/ml actinomycin D (Sigma-Aldrich St. Louis, Mo, USA) for 1h at 37°C the cells were than fixed and stained. Cells were washed with PBS, fixed for 15 min with 4% paraformaldehyde (Sigma-Aldrich St. Louis, Mo, USA), washed with PBS, permeabilized with 0.5% Triton X-100 for 10 min and blocked for 40 min with 0.2% gelatin. The incubation with mouse monoclonal antibody anti-B23 (ab10530, Abcam diluted at 1:200) was performed in PBS-Triton X-100 overnight at 4° C. Cells were than washed 3 x 5 min and incubated with secondary antibodies, Alexa Fluor mouse 594 diluted 1:1000. The analysis was performed by immunofluorescence microscopy (LEICA DM4000B).

**PCR (Polymerase chain reaction)**

ChIP samples were analysed by standard PCR in a final volume of 50 μl: 1× Buffer, 1.5 mM MgCl_2_, 100 μM dNTPs mixture, 1 μl of RBC Taq Polymerase (RBC Bioscience). Primers were used in a concentration of 0.5 μM are: B23pFW 5’-TCGAGGTGCTCTGGCTC -3’; B23pREV5’-TGCATAATGGCGTCGGCAG-3’. PCR amplification was performed as follows: one cycle of 5 min at 95 °C, followed by 40 cycles consisting of denaturation at 94 °C for 30 s, annealing at 55 °C for 30 s and elongation at 72 °C for 45 s; final elongation was at 72 °C for 10 min (My Clycler-Thermocycle Biorad). Subsequently, 20 μL of each PCR product were then fractionated in 2% agarose (Sigma) gel stained with ethidium bromide solution (10 mg/ml) and specific band was observed under UV light exposure.
